# Endothelial deletion of adipose triglyceride lipase protects against heart failure with preserved ejection fraction

**DOI:** 10.1172/jci.insight.187145

**Published:** 2025-03-10

**Authors:** Juliane Schwanbeck, Max Stahnke, Anna Eberlein, Madeleine Goeritzer, Arndt Schulze, Dominique Pernitsch, Dagmar Kolb, Gernot F. Grabner, Theda U.P. Bartolomaeus, Sofia K. Forslund, Holger Gerhardt, Gabriele G. Schiattarella, Lucia Cocera Ortega, Natalia López-Anguita, Erin E. Kershaw, Henrike Maatz, Norbert Hübner, Rudolf Zechner, Anna Foryst-Ludwig, Ulrich Kintscher

**Affiliations:** 1 Institute of Pharmacology, Charité – Universitätsmedizin Berlin,Berlin, Germany.; 2 DZHK (German Centre for Cardiovascular Research), Partner Site Berlin, Germany.; 3 Gottfried Schatz Research Center, Medical University of Graz, Austria.; 4 Experimental and Clinical Research Center, Charité/Max Delbrück Center for Molecular Medicine (MDC), Berlin, Germany.; 5 MDC, Berlin, Germany.; 6 Charité – Universitätsmedizin Berlin, Germany.; 7 Deutsches Herzzentrum der Charité (DHZC), Berlin, Germany.; 8 Division of Endocrinology and Metabolism, Department of Medicine, University of Pittsburgh, Pennsylvania, USA.; 9 Helmholtz Institute for Translational AngioCardioScience (HI-TAC) of the MDC at Heidelberg University, Germany.; 10 University of Graz, Institute of Molecular Biosciences, Graz, Austria.

**Keywords:** Cardiology, Endothelial cells, Heart failure, Mouse models

## To the Editor:

Heart failure with preserved ejection fraction (HFpEF) pathophysiology is multifactorial, with alterations in cardiac lipid metabolism likely playing a central role (1). Cardiac lipid metabolism is governed by transcellular uptake and transport of circulating lipids across cardiac endothelial cells (EC). In HFpEF, however, the processes of endothelial lipid handling and their functional relevance for lipid handling in the myocardium are largely unknown. Lipid processing in ECs relies on the function of the lipid droplet–hydrolyzing (LD-hydrolyzing) enzyme adipose triglyceride lipase (ATGL), the deletion or pharmacological blockade of which results in the accumulation of LDs in ECs (2–5).

**Figure 1:**
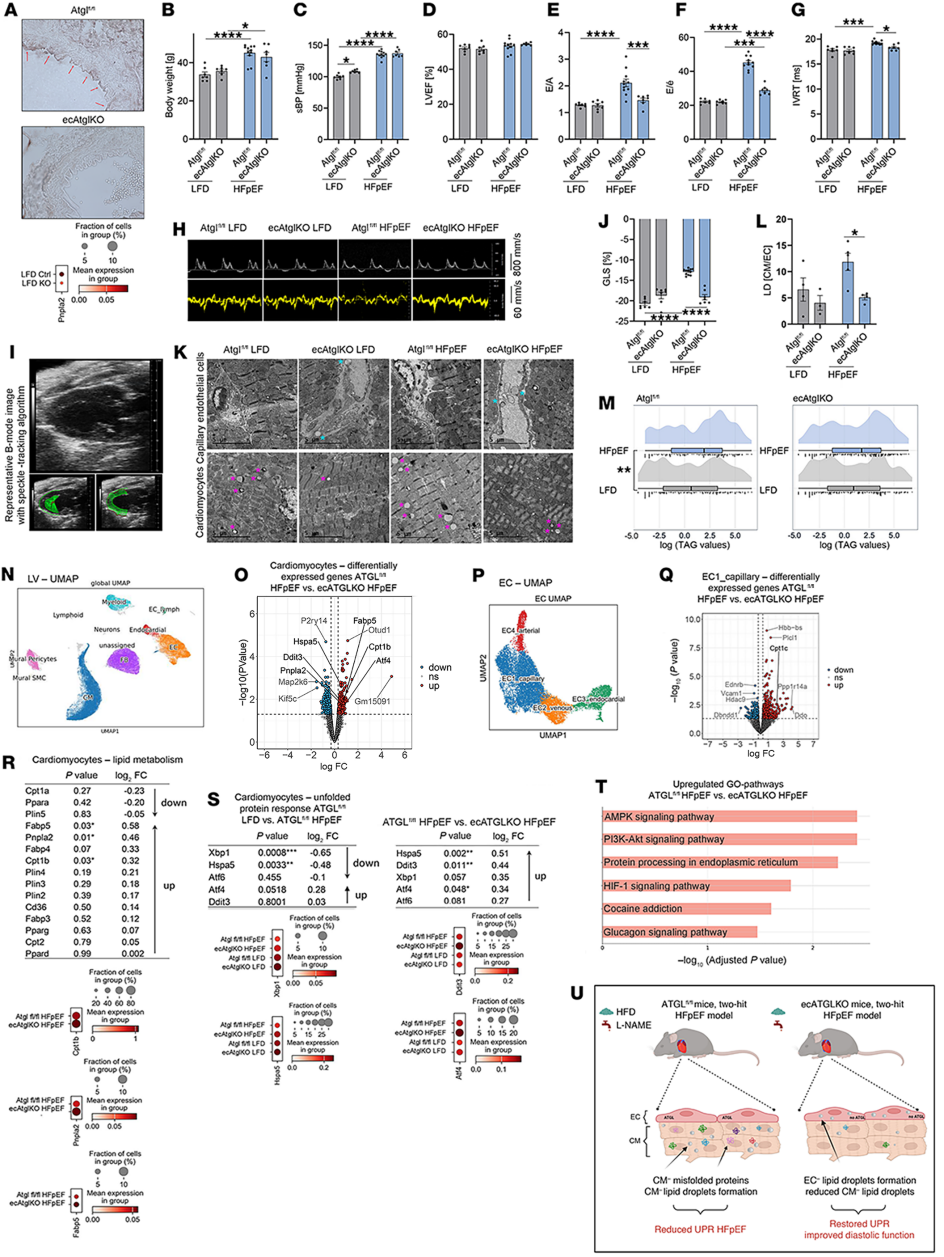
Endothelial deletion of ATGL protects against HFpEF. (**A**) Representative IHC of aortas with ATGL (brown, marked by arrows) (top). Reduced expression (single nuclei RNA-Seq, LV) of Pnpla2 (Atgl) in ECs (*n* = 4; *P* < 0.001) (bottom). (**B** and **C**) Body weight (BW) and systolic blood pressure (sBP) after 15 weeks. (**D**–**J**) Echocardiographic parameters; Atgl^fl/fl^-LFD (*n* = 7), ecAtglKO-LFD (*n* = 7), Atgl^fl/fl^-HFpEF (*n* = 11), and ecAtglKO-HFpEF (*n* = 7) mice (2-way ANOVA with Bonferroni’s/Tukey’s multiple-comparison tests). Echocardiographic analysis of Atgl^fl/fl^ LFD (*n* = 7), ecAtglKO LFD (*n* = 7), Atgl^fl/fl^ HFpEF (*n* = 11), and ecAtglKO HFpEF (*n* = 7) mice (2-way ANOVA with Bonferroni’s multiple comparison tests). (**D**) LV-ejection fraction (LVEF). (**E**–**G**) Parameters of diastolic dysfunction: E/A, E/e’, IVRT. (**H**) Representative pulse wave (top) and tissue Doppler (bottom) images. (**I**) Representative B-Mode images with or without speckle-tracking algorithm. (**J**) Global longitudinal strain (GLS). (**K**) Representative transmission electron micrographs (TEM) from LVs (cyan asterisks indicate LDs in ECs; purple asterisks indicate LDs in cardiomyocytes [CM]). (**L**) LD quantification shown as CM/EC ratio (2-way ANOVA/Bonferroni’s multiple comparison tests). (**M**) Triacylglycerol (TAG) content of LVs (*n* = 4) (Wilcoxon test). (**N**–**T**) Single nuclei RNA-Seq data from LV from Atgl^fl/fl^ and ecAtglKO mice (LFD/HFpEF, *n* = 4 per group). (**N**) Global UMAP of all cell types present in LVs of Atgl^fl/fl^-LFD/HFpEF and ecAtglKO-LFD/HFpEF mice. (**O**) Volcano plot of differentially expressed genes (DEGs, *P* < 0.05; log_2_FC > 0.3). (**P**) UMAP of the EC cluster. (**Q**) Volcano plot of DEGs in capillary ECs (DEGs, *P* < 0.05; log_2_FC > 0.3). (**R**) DEGs for lipid metabolism in CM (top), significant DEGs with *P* < 0.05 as dot plots (bottom) (Atgl^fl/fl^-HFpEF versus ecAtglKO-HFpEF mice). (**S**) DEGs characteristic for UPR in CMs: Atgl^fl/fl^-LFD versus Atgl^fl/fl^-HFpEF (left), Atgl^fl/fl^-HFpEF versus ecAtglKO-HFpEF (right). (**T**) GO term analysis of genes upregulated in cardiomyocytes from ecAtglKO-HFpEF versus Atgl^fl/fl^-HFpEF mice. (**U**) Graphical representation of study. *P < 0.05, ***P < 0.001, ****P < 0.0001.

EC-specific Atgl-KO mice (ecAtglKO) and their Cre WT littermate controls (Atgl^fl/fl^) (Figure 1A) were subjected to the 2-hit (high-fat diet [HFD]/L-NAME) HFpEF protocol (Figure 1, B and C) (6). Echocardiography after 15 weeks revealed a preserved left ventricular (LV) ejection fraction (EF) and LV hypertrophy in both genotypes (Figure 1D and Supplemental Figure 1, A–C; supplemental material available online with this article; https://doi.org/10.1172/jci.insight.187145DS1). Surprisingly, ecAtglKO mice were protected against HFD/L-NAME–mediated diastolic dysfunction (Figure 1, E–H) and exhibited improved global longitudinal strain (GLS) (Figure 1, I and J, and Supplemental Figure 1, D and E).

HFpEF was associated with enhanced LD formation in cardiomyocytes with few LDs detected in capillary ECs (Figure 1, K and L). Endothelial ATGL deletion led to enhanced LD formation in ECs and a reduced LD cardiomyocyte/EC ratio (Figure 1, K and L). LV triacylglycerol (TAG) accumulation significantly increased in Atgl^fl/fl^-HFpEF mice but not in ecAtglKO-HFpEF mice (Figure 1M and Supplemental Figure 1F). Distinct differences in mitochondrial or sarcomeric structure were not detected between Atgl^fl/fl^-HFpEF and ecAtglKO-HFpEF mice (data not shown).

We next performed single nuclei RNA-Seq from LV samples (Figure 1, N–Q, and Supplemental Figure 2, A and B). Comparison of Atgl^fl/fl^-HFpEF and ecAtglKO-HFpEF mice revealed 264 genes significantly upregulated and 261 downregulated in cardiomyocytes as well as 475 upregulated and 165 downregulated genes in capillary ECs (Figure 1, O and Q). We could not detect any clear inflammatory response in EC subclusters (Supplemental Figure 2, C and D, and Supplemental Figure 3). In cardiomyocytes from ecAtglKO-HFpEF mice, genes involved in FA metabolism were significantly upregulated (Figure 1R). Dysregulation of the unfolded protein response (UPR) has been recently identified as a pathogenic driver of HFpEF connected to lipid metabolism (6). Similarly, we found a significant reduction of cardiomyocyte genes involved in the IRE1α/XBP1 signaling pathway of the UPR in Atgl^fl/fl^-HFpEF mice (Figure 1S, left), accompanied by a reduction of IREα phosphorylation (Supplemental Figure 4A) (6). This reduction was notably absent in ecAtglKO-HFpEF mice, and genes involved in protein processing in the ER were upregulated (Figure 1, S [right] and T, and Supplemental Figure 4A). Finally, pharmacological inhibition of ATGL in ECs resulted in increased LD formation in ECs and reduced LD detection in cardiomyocytes, associated with increased expression of Hspa5/BiP in cardiomyocytes (Supplemental Figure 4, B–D).

In conclusion, endothelial-specific deletion of ATGL improved diastolic function in HFpEF accompanied by changes of neutral lipid storage at the capillary EC–cardiomyocyte interface. Mechanistically, reduced LD accumulation in cardiomyocytes may reverse the suppression of the IRE1α/XBP1 axis of the UPR (Figure 1U) (6). In addition to the effects observed at the capillary EC–cardiomyocyte interface, systemic metabolic effects may have contributed to the HFpEF improvement in ecAtglKO mice. Recent publications demonstrate that endothelial ATGL deletion promotes endothelial dysfunction, arterial hypertension, and atherosclerosis mediated by the suppression of the eNOS/NO pathway (2–5). The unique property of our model is that this pathway was continuously blocked by L-NAME, which likely prevented the detection of detrimental effects of LD formation on NO-dependent function and contributed to the phenotype observed in ecAtglKO-HFpEF mice. Finally, it is important to note that the 2-hit HFpEF model used here also has limitations and that an analysis of endothelial ATGL in other HFpEF models would be recommended.

## Supplementary Material

Supplemental data

Supporting data values

## Version 1 03/10/2025

Electronic publication
