# Supplementary material for: Endothelial deletion of adipose triglyceride lipase protects against heart failure with preserved ejection fraction
Source: JCI Insight. 2025 Mar 10;10(5):e187145. doi: 10.1172/jci.insight.187145 (PMC11949046; doi:10.1172/jci.insight.187145)
Supplement: Supplemental data [file jciinsight-10-187145-s311.pdf]

Supplemental Material

Endothelial Deletion of Adipose Triglyceride Lipase Protects Against Heart Failure with Preserved Ejection Fraction

Juliane Schwanbeck[1,2]*, Max Stahnke[1,2]*, Anna Eberlein[1,2], Madeleine Goeritzer[1,2], Arndt Schulze[1,2], Dominique Pernitsch[3], Dagmar Kolb[3], Gernot F. Grabner[3], Theda U.P. Bartolomaeus[2,4,5,6], Sofia K. Forslund[2,4,5,6], Holger Gerhardt[2,5], Gabriele G. Schiattarella[2,5,7], Lucia Cocera Ortega[2,5], Natalia López-Anguita[2,5], Erin E. Kershaw[8], Henrike Maatz[2,5], Norbert Huebner[2,5,6,9], Rudolf Zechner[10], Anna Foryst-Ludwig[1,2]*, Ulrich Kintscher[1,2]*

[1] Charité – Universitätsmedizin Berlin, Institute of Pharmacology, Berlin, Germany

[2] DZHK (German Centre for Cardiovascular Research), Partner Site Berlin, Germany

[3] Gottfried Schatz Research Center, Medical University of Graz, Austria

[4] Experimental and Clinical Research Center, Charité/ Max Delbrück Center for Molecular Medicine (MDC), Berlin, Germany

[5] MDC, Berlin, Germany

[6] Charité – Universitätsmedizin Berlin, Germany

[7] Deutsches Herzzentrum der Charité (DHZC), Berlin, Germany

[8] Division of Endocrinology and Metabolism, Department of Medicine, University of Pittsburgh, PA, USA

[9] Helmholtz Institute for Translational AngioCardioScience (HI-TAC) of the MDC at Heidelberg University, Germany

[10] University of Graz, Institute of Molecular Biosciences, Graz, Austria

*authors contributed equally; the authors have declared that no conflict of interest exists

Methods

Sex as a biological variable

Our study examined male mice because female animals develop a significantly attenuated phenotype in the used two-hit mouse model of HFpEF (1).

Animals

Atgl-floxed (Atgl$^{fl/fl}$) mice (B6.129-Pnpla2tm1Eek) were cross-bred with endothelial cell-specific Cdh5 CreERT2 mice to generate mice with an inducible endothelial cell-specific Atgl-knockout. 6–8-week-old male mice were kept in a temperature- and light-controlled facility with a 12 h light/dark cycle, randomized by body weight into four groups (Atgl$^{fl/fl}$ LFD (n=7), ecAtglKO LFD (n=7), Atgl$^{fl/fl}$ HFpEF (heart failure with preserved ejection fraction) (n=11), and ecAtglKO HFpEF(n=7)), and caged in groups of 2-4 animals per cage. To develop HFpEF mice were subjected to a hypertensive-obese two–hit HFpEF model described previously (2). Briefly, mice received either LFD (10 % energy from fat, Research diets) and drinking water or HFD (60 % energy from fat, Research diets) and N$^{\omega}$-nitro-L-arginine methyl ester (L-NAME, 0.5 g/L, Sigma Aldrich; pH 7.4) in drinking water ad libitum. After 15 weeks of intervention mice were phenotyped and sacrificed under isoflurane anesthesia by cervical dislocation. Blood and tissue samples were collected for further analysis.

Echocardiography

Cardiac function was assessed by echocardiography 15 weeks after starting HFD/ L-NAME intervention as previously described (3) Echocardiographic measurements were conducted using a Vevo 3100 Imaging System equipped with a 30-MHz linear transducer. Animals were initially anesthetized with 3 % isoflurane, which was subsequently reduced to 1-1.5 % for image acquisition to maintain consistent heart rates. For image evaluation, the Vevo Lab analysis software (FUJIFILM VisualSonics Inc., Canada) was used. Systolic function and cardiac dimensions were analyzed using B-Mode and M-Mode images obtained from the parasternal long axis and the parasternal short axis at the mid-papillary level. Diastolic function was

investigated using conventional pulsed wave Doppler to measure transmitral flow parameters in the apical four chamber view. Tissue Doppler images recorded from the septal anulus of the mitral valve were analyzed to assess myocardial relaxation velocity. The following parameters were analyzed: E/A: early diastolic flow velocity/ active atrial contraction; E/e': early diastolic flow velocity/ velocity of early diastolic mitral annular motion; IVRT: isovolumetric relaxation time. Strain and M-Mode analysis involved the assessment of three images with three cardiac cycles for each image, that were subsequently averaged. Diastolic measurements were performed using three different doppler images with three measurements each. Parasternal long axis and short axis images were used for Speckle Tracking analysis, with vectors of the epicardial and endocardial borders used for analysis or longitudinal, circumferential and radial deformation.

Blood Pressure Analysis

Systolic blood pressure was determined using the tail-cuff method (IITC Life Science, Woodland Hills, CA, USA) following the 15-week HFD/ L-NAME intervention. To ensure reliable data collection and minimize stress, mice underwent a three-day training period prior to the final measurement, during which they acclimated to the restrainer. To familiarize them with the environment, mice remained in the chamber for 5 minutes before measurements started. Blood pressure was monitored during the relaxation phase subsequently to tail artery constriction, with at least 10-15 recordings obtained per animal per session. Systolic blood pressure was then calculated as the mean of multiple measurements per mouse.

Electron Microscopy

Cardiac muscle tissue was performed according to a previously published protocol (4). Ultrathin sections (70nm) were cut with a UC 7 Ultramicrotome (Leica Microsystems, Vienna, Austria) and stained with lead citrate for 5 min and platin blue for 15 min. Electron micrographs were taken using a Tecnai G2 transmission electron microscope (FEI, Eindhoven, Netherlands) with a Gatan ultrascan 1000 charge coupled device (CCD) camera (−20 °C;

acquisition software Digital Micrograph; Gatan, Munich, Germany). Acceleration voltage was 120 kV. Further investigations were made with the Scanning electron microscope. The Scanning transmission electron microscopy mode (STEM) of a field emission scanning electron microscope (ZEISS FE-SEM Sigma 500) in combination with ATLAS TM was used to perform imaging on large areas of cardiac muscle tissue with high resolution. LDs were counted manually.

Immunostaining

For immunohistochemical staining, tissue samples were initially deparaffinized in Neo-Clear for 5 minutes, followed by sequential immersion in 100%, 96%, 80%, and 70% ethanol solutions, as well as bidest, each for 2 minutes. Subsequently, the sections were subjected to heat-induced antigen retrieval in preheated citrate buffer, heated for 5 minutes at 600 W in a microwave oven. After the addition of 50 mL of H_2O, the samples underwent an additional 5-minute heat treatment at 600 W. The sections were then cooled to room temperature, rinsed in phosphate-buffered saline (PBS), and incubated in 0.3% H_2O_2/methanol solution for 30 min. After rinsing again with PBS, the sections were incubated with 10% blocking serum (Normal-Goat Serum) for 30 minutes, followed by overnight incubation at 4 °C with the ATGL antibody. The next day, after three PBS washes, the sections were incubated with the secondary antibody for one hour at room temperature. Subsequently, the sections were treated with the DAB reagent for 3 minutes at room temperature. After a brief washing step, the samples were counterstained with Hemalaun for 5 seconds and fixed with Hydromount.

TAG quantification in heart tissue

TAGs were measured in heart tissue harvested from $Atgl^{fl/fl}$ LFD, ecAtglKO LFD, $Atgl^{fl/fl}$ HFpEF, and ecAtglKO HFpEF mice, as described previously (5). 20 mg of cardiac tissue samples were pulverized in liquid nitrogen and used for lipid extraction according to Folch’s protocol. The extracts were dried under a stream of nitrogen at 40 °C. The samples were next reconstituted with 100 µl EtOH/ 100 µL of 40 nM deuterated internal standard (d5-TG Internal Standard

Mixture I, Avanti Polar Lipids, Alabaster, AL). LC/MS analysis was performed using Agilent 1290 HPLC coupled with a 6470 Triplequadrupole mass spectrometer (Agilent Technologies, Santa Clara, CA) in positive electrospray mode. A Zorbax Eclipse plus C18 50 x 3 mm, 1.8 µm was used as stationary phase. Mobile phase was a gradient of 95 % to 100 % methanol and water mixed with 5 mM Ammonium acetate. 52 mass transitions were detected for individual TAG species and quantified using deuterated standards.

Western Blotting

Western blotting was performed as previously described (5). Briefly, cardiac tissue samples were lysed in RIPA buffer (50 mM Tris, pH 7.5, 150 mM NaCl, 5 mM MgCl2, 1 % Nonidet P-40, 2.5 % glycerol, 1 mM EGTA, 50 mM NaF, 1 mM Na3VO4, 10 mM Na4P2O7, 100 M phenylmethylsulfonyl fluoride, and protease inhibitors (Complate TM, Roche Diagnostics)). Lysates were separated by 12 % SDS-PAGE gels and blotted onto a PVDF-Membrane. Proteins were detected using rabbit anti-pIRE1a antibody (48287, Cell Signaling, United Kingdom), rabbit anti-pIRE1 antibody (37073, Cell Signaling, United Kingdom) and respective horseradish peroxidase-coupled secondary antibodies (711-035-152, Jackson ImmunoResearch, United States). For detection, enhanced chemiluminescent reagents (ECL Western Blotting Reagents, GE Healthcare, United States) were used. Signal densities were analyzed using Image Lab software (Bio-Rad, version 6.0.1).

Endothelial cell – Cardiomyocyte Co-Culture Experiments

Co-culture experiments were performed using human umbilical vein endothelial cells (HUVECs) (Promocell, Germany) seeded in Transwells (0.4 µM pore size from Corning) at a density of 2.5 x 10^5 per Transwell. Simultaneously, HL-1 cells were seeded at 4.5 x 10^5 cells per well in gelatin-fibronectin precoated 6-well glass plates from Cellvis. Both cell types were given 24 hours to grow. Afterwards, HL-1 cells were starved for 1 hour in HL-1 starving medium, composed of 0.5% FBS, 1% P/S, and 1% L-Glutamine in basal Claycomb medium (Sigma-Aldrich). The HUVECs in Transwells were also starved for 1 hour with EC MV2

starving medium, which contained 0.5% EC Supplement Mix and 1% P/S in basal EC MV2 growth medium (Promocell, Germany). Following starvation, HUVECs were incubated with NG-497 (20 µM, dissolved in DMSO, ATGL Inhibitor) (6) or DMSO in Transwells for 1 hour. For the fatty acid (FA) stimulation, a mix of linoleic acid (18:2, Cayman, 225 µM) and oleic acid (18:1, Cayman, 150 µM) bound to Bovine Serum Albumin (BSA, 100 mg/ml) was prepared in EC MV2 starving medium. As control a mix of Ethanol (ETOH) and BSA (100 mg/ml) was prepared in the same manner as the FAs. NG-497 (20 µM) or DMSO was added to the FA and Ethanol/BSA mix. Next HL-1 cells were provided with Claycomb starving medium supplemented with 0.5% FA-free BSA per well. The EC MV2 starving medium was removed and the cells were co-cultured. At last, the FA mix or ETOH BSA control mix with either DMSO or NG-497 (20 µM) was added to the Transwells. The stimulation period and co-culture lasted for 16 hours.

Immunofluorescence

After 16 hours of FA stimulation, HUVECs and HL-1 cells were fixed with 2% paraformaldehyde (PFA) for 10 minutes in 6-well glass plates or Transwells. BODIPY 493/503 staining was performed on both cell types using a 10 µg/mL solution for 15 minutes at room temperature. Next, the cells were permeabilized with 0.1% Triton X-100 in 1X PBS for 10 minutes at room temperature. For Nuclei staining, DAPI was used in a concentration of 1 µg/mL for 7 minutes at room temperature. In between washing steps with 1X PBS were included. After staining, the cells were kept in 1x PBS for imaging. Imaging was conducted using a Zeiss Axio Observer microscope. Images were captured using 10x for Transwells and 20x and 40x magnification for 6-well glass plates with consistent exposure times for the green and blue channel across all images.

Quantitative Real Time PCR

qRT-PCR analysis was performed as previously described (5). Briefly, after co-culture both cell types HUVECs and HL-1 cells (12 Samples each group) were lysed and total RNA was extracted from cells using RNeasy Mini kit according to the manufacture's protocol (Qiagen, Germany). cDNA was synthesized by reverse transcription using reverse transcriptase, RNAsin and dNTPs (all Promega, United States). For gene expression quantification real-time quantitative polymerase chain reaction (RT-qPCR) was performed on a CFX96 Real-Time PCR System (BioRad, United States) using the SYBR-Green technology. Relative gene expression was calculated by 2-ΔΔCT method with ppia (peptidylprolyl Isomerase A, HL-1) or β-Actin (long-term treatment effect subgroup, HUVECs) as housekeeping gene. For the qRT-PCR analysis we used primers sequences listed below:

HL-1 cells: ppia: CCGATGACGAGCCCTTGG and GTAAAGTCACCACCCTGGCAC; hsp5a: TGTGTGAGACCAGAACCGTC and GAACACACCGACGCAGGAAT; HUVECs: β-Actin: GGGTCAGAAGGATTCCTATG and GGTCTCAAACATGATCTGGG, FABP4: ACTGGGCCAGGAATTTGACG and CTCGTGGAAGTGACGCCTT, CD36: AgATgCAgCCTCATTTCCAC and CgTCggATTCAAATACAgCA.

Single-nucleus RNA-seq (snRNA-seq) analysis

Isolation of Single nuclei from cardiac left ventricle samples and processing using the 10x Genomics platform

Nuclei isolation and library preparation were conducted at the Max-Delbrück Center for Molecular Medicine, Berlin, Germany, as previously described (7, 8). In brief, DAPI stained and FACS sorted isolated nuclei from flash-frozen tissue underwent visual inspection under a microscope to assess integrity and were manually or automatically counted using a Countess II (Life Technologies). The nuclei suspension was then loaded onto the Chromium Controller (10X Genomics) with a targeted recovery of 5,000 nuclei per reaction. 3′ gene expression libraries were prepared using the v3 Chromium Single Cell Reagent Kits (10X Genomics) following the manufacturer's instructions. Quality control of the final library cDNA was

performed using a Bioanalyzer High Sensitivity DNA Analysis (Agilent) and the KAPA Library Quantification kit. The libraries were sequenced on an Illumina NovaSeq, with a target of 30,000-50,000 reads per nucleus.

Data pre-processing and read mapping

Following demultiplexing and conversion of Bcl to Fastq files using bcl2fastq, each sample was aligned to the mouse reference genome GRCm38 with a modified pre-mRNA GTF file from Ensembl release Ens98 using the CellRanger suite (v.6.1.2) with default parameters. Reads that mapped within both exonic and intronic regions were counted, and mapping quality was assessed using CellRanger summary statistics. Reads that overlapped multiple sequence features were discarded.

Quality control, batch correction and clustering

For quality control and downstream analysis, the Python Scanpy v1.9.0 toolkits were used as described before (8-10). Briefly, doublets were identified and filtered out using Solo (Solo soft max scores ≤ 0.5) (11). Single nuclei were filtered based on counts (300 ≤ n_counts ≤ 15,000), genes (300 ≤ n_genes ≤ 5,000), mitochondrial genes (percent_mito ≤ 1%), and ribosomal genes (percent_ribo ≤ 1%). Following read count normalization and log-transformation, highly variable genes were selected. The effects of mitochondrial gene percentage and total counts per nucleus were regressed out, and the resulting values were scaled to unit variance. Principal components were computed, and elbow plots used to determine the optimal number of principal components for neighbor graph construction. The selected principal components were adjusted using Harmony (12). Dimensionality reduction was performed using the Uniform Manifold Approximation and Projection method (UMAP) and Leiden clustering was used to identify cellular communities (13). Doublets are colored light grey and excluded from further analysis. To identify genes differentially expressed (DEG) between clusters that served as marker genes to allow manual annotation of cell types and states, the Wilcoxon rank sum test was used as implemented in Scanpy. Testing for DEGs was done using log-transformed and

normalized to library size count values. Only genes with mean expression > 0.0125 were considered. Cell types were subsequently sub-clustered to identify clusters with chimeric transcriptional profiles that persisted despite Solo-based doublet removal. These RNA-loaded droplets may represent real biology, background RNA noise, or multiplets and were therefore labelled as unassigned. To identify distinct cell states, sub-clusterings were then repeated excluding unassigned droplets.

Genotype-specific differential gene expression analysis

To identify genotype-specific expression profiles in cardiomyocytes and endothelial cells, transcript counts per gene of all nuclei for a given cluster (cell type level) or sub-cluster (state level) from the same animal were aggregated to create "pseudo-bulk" samples. Testing for DEGs in pseudo-bulks was performed using the empirical Bayes quasi-likelihood F-tests function (glmQLFtest) available in the R package EdgeR (version 3.28.1) (14, 15) Genes with a p-value < 0.05 and a |log2FC| > 0.3 were defined as differentially expressed.

Gene ontology (GO) enrichment analysis

GO analysis was performed using GSEApy v0.10.5 – a Python implementation for Enrichr with default settings (16). GO analyses were performed with the gene-set libraries "KEGG_2019_Mouse" and DEGs identified in cardiomyocytes were used as input (|log2FC| > 0.3 & p-value < 0.05). Gene background was defined using all genes that were expressed in cardiomyocytes (mean expression > 0.0125).

Statistical Analysis

Statistical analysis was performed using GraphPad Prism 8.3.0 software. Results are presented as mean ± SEM. Two-way ANOVA with Tukey or Bonferroni multiple comparisons was performed for multiple-group analysis. For two-group analysis, Wilcoxon test was used.

For analyses of snRNA-seq data, Wilcoxon rank sum test and Benjamini Hochberg correction were used to identify cell type and cell state specific marker genes. For differential expression

analysis, glmQLFTest and Benjamini Hochberg correction, implemented in EdgeR, were applied.

Study approval

Animal experiments were performed according to the German Animal Welfare Act and approved by the State Office for Health and Social Affairs Berlin, Germany (LAGeSo).

Data reporting

Single nuclei RNA sequencing raw data for all samples including the two genotypes (Atgl fl/fl; ecAtgl/KO) have been deposited in the European Nucleotide Archive (ENA) at EMBL-EBI under accession number PRJEB72644:

(https://www.ebi.ac.uk/ena/browser/view/PRJEB72644)

Data values for all graphs, and values behind any reported means in the manuscript or supplement are documented in the supporting data values XLS file.

The graphical abstract was created with BioRender.

References

1. Tong D, Schiattarella GG, Jiang N, May HI, Lavandero S, Gillette TG, et al. Female Sex Is Protective in a Preclinical Model of Heart Failure With Preserved Ejection Fraction. *Circulation.* 2019;140(21):1769-71.
2. Schiattarella GG, Altamirano F, Tong D, French KM, Villalobos E, Kim SY, et al. Nitrosative stress drives heart failure with preserved ejection fraction. *Nature.* 2019;568(7752):351-6.
3. Beyhoff N, Brix S, Betz IR, Klopfleisch R, Foryst-Ludwig A, Krannich A, et al. Application of Speckle-Tracking Echocardiography in an Experimental Model of Isolated Subendocardial Damage. *J Am Soc Echocardiogr.* 2017;30(12):1239-50 e2.
4. Galhuber M, Kupper N, Dohr G, Gauster M, Kwapiszewska G, Olschewski A, et al. Simple method of thawing cryo-stored samples preserves ultrastructural features in electron microscopy. *Histochem Cell Biol.* 2021;155(5):593-603.
5. Thiele A, Luettges K, Ritter D, Beyhoff N, Smeir E, Grune J, et al. Pharmacological inhibition of adipose tissue adipose triglyceride lipase by Atglistatin prevents catecholamine-induced myocardial damage. *Cardiovascular research.* 2022;118(11):2488-505.
6. Grabner GF, Guttenberger N, Mayer N, Migglautsch-Sulzer AK, Lembacher-Fadum C, Fawzy N, et al. Small-Molecule Inhibitors Targeting Lipolysis in Human Adipocytes. *J Am Chem Soc.* 2022;144(14):6237-50.
7. Nadelmann ER, Gorham JM, Reichart D, Delaughter DM, Wakimoto H, Lindberg EL, et al. Isolation of Nuclei from Mammalian Cells and Tissues for Single-Nucleus Molecular Profiling. *Curr Protoc.* 2021;1(5):e132.
8. Reichart D, Lindberg EL, Maatz H, Miranda AMA, Viveiros A, Shvetsov N, et al. Pathogenic variants damage cell composition and single cell transcription in cardiomyopathies. *Science.* 2022;377(6606):eabo1984.
9. Hao Y, Hao S, Andersen-Nissen E, Mauck WM, 3rd, Zheng S, Butler A, et al. Integrated analysis of multimodal single-cell data. *Cell.* 2021;184(13):3573-87 e29.
10. Wolf FA, Angerer P, and Theis FJ. SCANPY: large-scale single-cell gene expression data analysis. *Genome Biol.* 2018;19(1):15.
11. Bernstein NJ, Fong NL, Lam I, Roy MA, Hendrickson DG, and Kelley DR. Solo: Doublet Identification in Single-Cell RNA-Seq via Semi-Supervised Deep Learning. *Cell Syst.* 2020;11(1):95-101 e5.
12. Korsunsky I, Millard N, Fan J, Slowikowski K, Zhang F, Wei K, et al. Fast, sensitive and accurate integration of single-cell data with Harmony. *Nat Methods.* 2019;16(12):1289-96.
13. Traag VA, Waltman L, and van Eck NJ. From Louvain to Leiden: guaranteeing well-connected communities. *Sci Rep.* 2019;9(1):5233.
14. Chen Y, Lun AT, and Smyth GK. From reads to genes to pathways: differential expression analysis of RNA-Seq experiments using Rsubread and the edgeR quasi-likelihood pipeline. *F1000Research.* 2016;5:1438.
15. Robinson MD, McCarthy DJ, and Smyth GK. edgeR: a Bioconductor package for differential expression analysis of digital gene expression data. *Bioinformatics.* 2010;26(1):139-40.
16. Fang Z, Liu X, and Peltz G. GSEApy: a comprehensive package for performing gene set enrichment analysis in Python. *Bioinformatics.* 2023;39(1).

Acknowledgments

We thank Beata Hoeft (Charité – Universitaetsmedizin Berlin, Germany) for her excellent technical assistance and Michael Rothe (Lipidomix GmbH, Berlin, Germany) for the lipid analysis.

This study was supported by grants from the Einstein Foundation/Foundation Charité, the Deutsche Forschungsgemeinschaft (DFG) and the German Centre for Cardiovascular Research (DZHK): UK is supported by the Einstein Foundation/ Foundation Charité (EVF-BIH-2018-440) and the DFG (DFG-KI 712/10-1 and SFB-1470-A09). JS, UK, and AFL are supported by the DZHK, BER 5.4 PR. SKF is supported by the DFG SFB-1470-A05. EEK and generation of the Atgl$^{fl/fl}$ model was supported by National Institutes of Health R01KD0960166. NH is supported by the DFG SFB-1470-B03, the Chan Zuckerberg Foundation, and an ERC Advanced Grant from the European Union. GGS is supported by DZHK 81X3100210 and 81X2100282, DFG (SFB-1470–A02 and SFB-1470–Z01) and European Research Council ERC StG 101078307.

Author contributions statement

JS: designing research studies, conducting experiments, acquiring data, analyzing data, writing the manuscript; MS: conducting experiments, acquiring data, analyzing data, revising the manuscript draft; AE: designing research studies, conducting experiments, acquiring data, analyzing data; MG: conducting experiments, acquiring data, analyzing data; AS: analyzing data, revising the manuscript draft; DP: analyzing data; DK: designing research studies, acquiring data, analyzing data; GFG: providing material, revising the manuscript draft; TUPB: analyzing data; SKF: designing research studies, analyzing data, revising the manuscript draft; HG: designing research studies, analyzing data, revising the manuscript draft; GGS: designing research studies, analyzing data, revising the manuscript draft; LCO: analyzing data; NLA: analyzing data, revising the manuscript draft; EEK: designing research studies, providing mice, revising the manuscript draft; HM: designing research studies, analyzing data, writing the manuscript; NH: designing research studies, analyzing data, writing the manuscript; RZ: designing research studies, providing material, providing funding, revising the manuscript draft; AFL: designing research studies, conducting experiments, acquiring data, analyzing data, writing the manuscript; UK designing research studies, providing funding, analyzing data, writing the manuscript.

Figure Legends (Supplemental Figures)

Fig. S1. A-E, Echocardiographic parameters of Atgl$^{fl/fl}$ LFD, ecAtglKO LFD, Atgl$^{fl/fl}$ HFpEF, ecAtglKO HFpEF mice (n = 7 - 11). **A**, LVEDD – left ventricular end diastolic diameter, **B**, LVAW – left ventricular anterior wall, **C**, LVPW – left ventricular posterior wall, **D**, GCS – global circumferential strain, **E**, GRS – global radial strain. **F**, Triglyceride content of left ventricle (n=4)

Fig. S2. A, Barcode rank plot across all samples, split and coloured by condition. Clear distinction between nuclei containing droplets and empty droplets (background ambient RNA) indicating an overall low background. **B**, Violin plots showing number of detected genes (n_genes), detected UMIs (n_counts), fraction of UMIs mapping to mitochondrial-encoded genes (percent mito) and ribosomal genes (percent_ribo) and doublet probabilities according to solo, per nucleus within a cell-type. **C,** GO term analysis of genes upregulated in capillary endothelial cells from ecAtglKO-HFpEF vs. Atgl$^{fl/fl}$-HFpEF mice. **D**, Differentially expressed genes (DEGs) characteristic for inflammation (red: pro-inflammatory, green: anti-inflammatory) in capillary endothelial cells from ecAtglKO-HFpEF vs. Atgl$^{fl/fl}$-HFpEF mice (n = 4) with Atglfl/fl HFpEF mice set as reference.

Fig. S3. A, Differentially expressed genes (DEGs) characteristic for inflammatory genes in the endothelial cell subcluster shown as dot blot. **B-D**, DEGs characteristic for inflammatory genes in different cell states of the endothelial cell subcluster. **B**, Atgl$^{fl/fl}$ HFpEF mice were compared to ecAtglKO HFpEF mice (n = 4) with Atgl$^{fl/fl}$ HFpEF mice set as reference. **C**, Atgl$^{fl/fl}$ LFD mice were compared to ecAtglKO LFD (n = 4) with Atgl$^{fl/fl}$ LFD mice set as reference. **D**, Atgl$^{fl/fl}$ LFD mice were compared to Atgl$^{fl/fl}$ HFpEF mice (n = 4) with Atgl$^{fl/fl}$ LFD mice set as reference. DEGs with p-Values < 0.05 are bold printed. *p<0.05, **p<0.01, ***p<0.001.

Fig. S4. A, Western Blot analysis of phospho- and total IRE1alpha in cardiac samples of Atglfl/fl LFD, ecAtglKO LFD, Atglfl/fl HFpEF, ecAtglKO HFpEF mice (n = 3). **B**, Experimental design of the ECs/CMs co-culture experiments. **C**, Immunofluorescence analysis of the co-culture experiments of ECs and CMs, stained with Bodipy 493/503 for LD formation and with DAPI for nuclear DNA, (N=3, n=3). Representative photos are shown. Exemplary LD are indicated with red arrows, with 40x magnification. **D**. qRT-PCR analysis of the FABP4, CD36 in ECs, and HSP5a in CM (N=3, n=3, ** p< 0.01). ECs: endothelial cells, CMs: cardiomyocytes, IRE1α: Endoplasmic Reticulum to Nucleus Signaling 1 alpha, FABP4: Fatty Acid Binding Protein 4, CD36: Fatty Acid Translocase, HSP5a: Heat Shock Protein Family A Member 5 alpha.

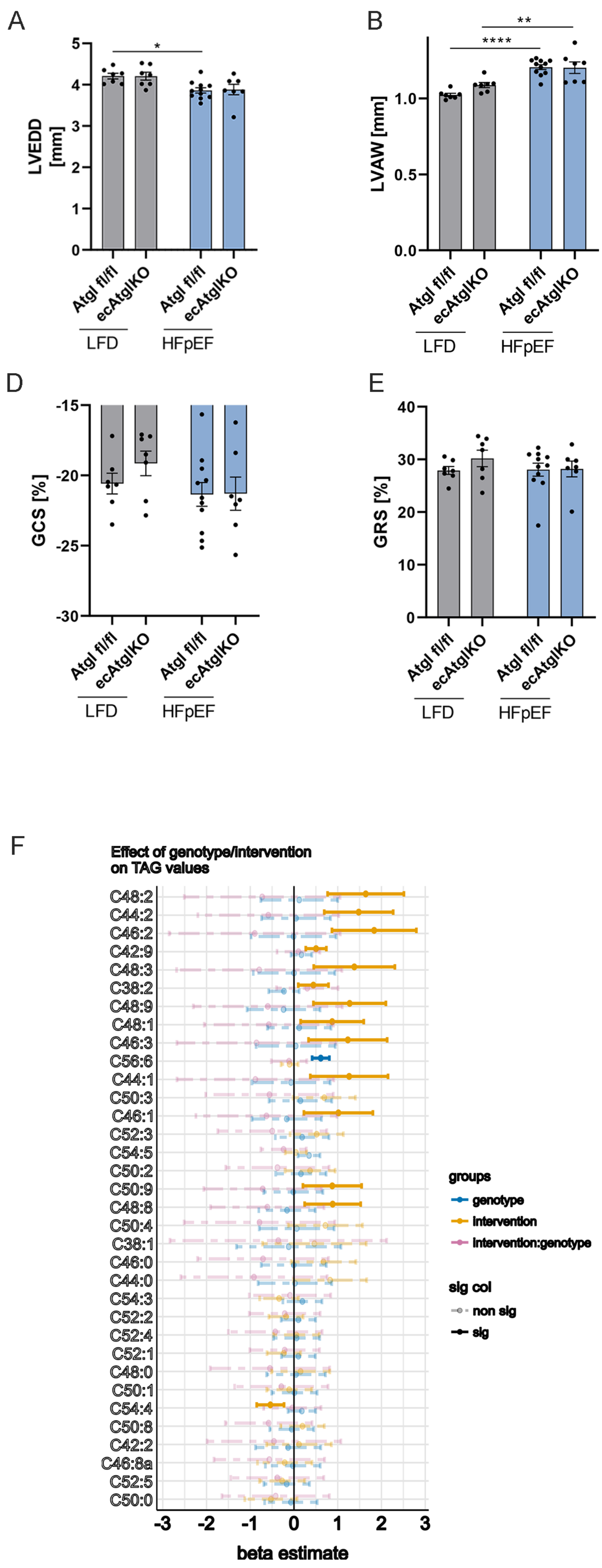
A
LVEDD [mm]
Atgl fl/fl
ecAtglKO
LFD
HFpEF
B
LVAW [mm]
C
LVPW [mm]
D
GCS [%]
E
GRS [%]
F
Effect of genotype/intervention on TAG values
beta estimate
groups
genotype
intervention
intervention:genotype
sig col
non sig
sig

Figure S1

A

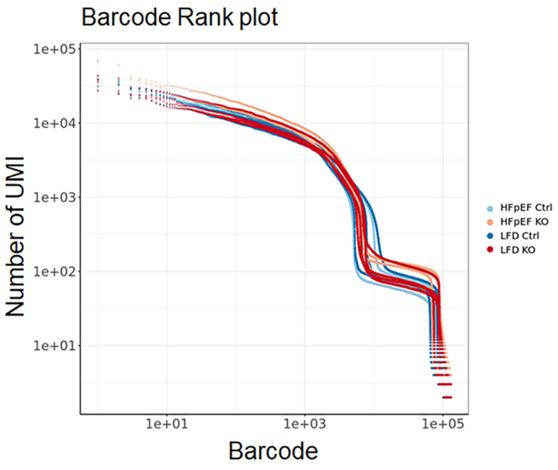

B

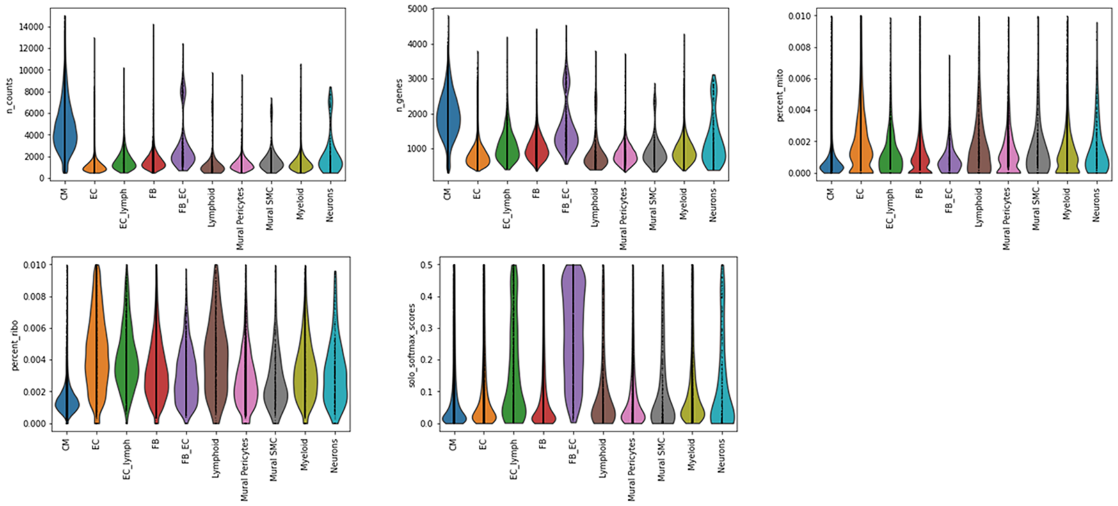

C

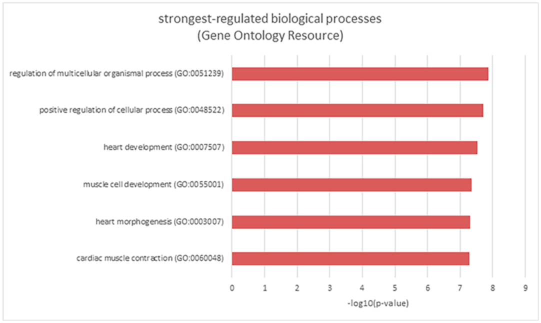

D

Atgl fl/fl HFpEF vs. ecAtglKO HFpEF

upregulated genes	logFC	PValue	FDR	full name
Cd2bp2	1,129	0,0083	0,430	CD2 Cytoplasmic Tail Binding Protein 2
Cxcr4	1,049	0,0153	0,543	C-X-C Motif Chemokine Receptor 4
Eda2r	0,624	0,0142	0,518	Ectodysplasin A2 Receptor
Ifit2	0,633	0,0092	0,445	Interferon Induced Protein With Tetratricopeptide Repeats 2
Nfatc2	3,022	0,0003	0,106	NFAT1
Tab1	0,966	0,0166	0,543	TGF-Beta Activated Kinase 1 (MAP3K7) Binding Protein 1
Tlr4	0,640	0,0073	0,419	Toll-Like Receptor 4
Tradd	0,981	0,0401	0,703	TNFRSF1A Associated Via Death Domain
Nlrc5	0,729	0,0020	0,279	NLR Family CARD Domain Containing 5
Smad6	0,522	0,0035	0,335	SMAD Family Member 6, i-SMAD (inhibitory)
Vsir	0,506	0,0288	0,633	V-Set Immunoregulatory Receptor

downregulated genes	logFC	PValue	FDR	full name
Ccl21a	-0,693	0,0039	0,335	C-C Motif Chemokine Ligand 21
Csf1	-0,583	0,0035	0,335	Colony Stimulating Factor 1,
Elp1	-0,569	0,0441	0,721	Elongator Acetyltransferase Complex Subunit 1
Il7	-1,540	0,0499	0,748	Interleukin 7
Msr1	-1,355	0,0492	0,748	Macrophage Scavenger Receptor 1
Ptger4	-0,514	0,0340	0,676	Prostaglandin E Receptor 4,
Pycard	-0,833	0,0162	0,543	PYD And CARD Domain Containing
Rnf25	-0,753	0,0087	0,441	Ring Finger Protein 25
Tgfb3	-1,814	0,0281	0,633	Transforming Growth Factor Beta 3
Vcam1	-0,858	0,0003	0,106	Vascular Cell Adhesion Molecule 1

Figure S2

A

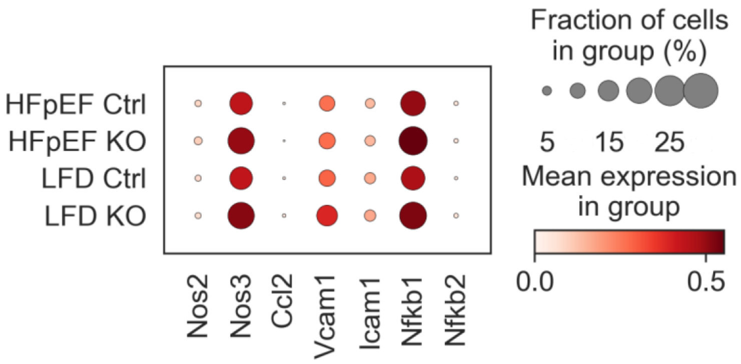

B

Atgl fl/fl HFpEF vs. ecAtglKO HFpEF								
**Inflammatory Genes**	**EC1_venous**		**EC2_capillary**		**EC3_endocardial**		**EC4_arterial**	
	**p-Value**	**log2FC**	**p-Value**	**log2FC**	**p-Value**	**log2FC**	**p-Value**	**log2FC**
**Nos2**	0.17	-0.74	0.24	0.27	0.23	2.39	0.20	-1.79
**Nos3**	0.98	-0.006	0.74	-0.06	0.79	0.06	0.51	-0.20
**Ccl2**	0.11	-1.56	0.08	-0.79	0.43	-1.22	0.65	-0.76
**Vcam1**	0.12	-0.3	**0.0003 *****	**-0.86**	0.69	-0.08	0.41	0.35
**Icam1**	0.43	-0.21	**0.04 ***	**-0.45**	0.66	-0.14	0.80	0.15
**Nfkb1**	0.51	-0.14	0.62	-0.08	0.50	0.16	0.98	-0.01
**Nfkb2**	0.49	-0.41	0.25	-0.38	0.78	0.16	0.77	-0.29

C

Atgl fl/fl LFD vs. ecAtglKO LFD								
**Inflammatory Genes**	**EC1_venous**		**EC2_capillary**		**EC3_endocardial**		**EC4_arterial**	
	**p-Value**	**log2FC**	**p-Value**	**log2FC**	**p-Value**	**log2FC**	**p-Value**	**log2FC**
**Nos2**	0.51	-0.42	0.73	-0.10	0.44	1.15	0.62	0.82
**Nos3**	0.52	0.14	0.89	-0.03	0.58	0.12	0.91	-0.04
**Ccl2**	0.45	0.48	**0.002 ****	**-1.67**	0.11	-1.80	0.46	0.74
**Vcam1**	**0.006 ****	**0.56**	0.68	-0.10	0.10	0.33	0.95	0.04
**Icam1**	0.77	0.08	0.85	-0.05	0.78	0.08	0.47	-0.48
**Nfkb1**	0.40	-0.19	0.30	0.19	0.67	-0.09	0.71	0.13
**Nfkb2**	0.13	1.04	0.08	0.69	0.78	-0.30	0.43	-1.23

D

Atgl fl/fl LFD vs. Atgl fl/fl HFpEF								
**Inflammatory Genes**	**EC1_venous**		**EC2_capillary**		**EC3_endocardial**		**EC4_arterial**	
	**p-Value**	**log2FC**	**p-Value**	**log2FC**	**p-Value**	**log2FC**	**p-Value**	**log2FC**
**Nos2**	0.60	0.29	0.68	-0.10	-	-	0.34	1.17
**Nos3**	0.08	0.34	0.13	0.25	1.00	0.001	0.71	0.11
**Ccl2**	0.75	-0.22	0.39	-0.31	0.89	-0.17	0.07	-2.07
**Vcam1**	1.01E+09	0.78	0.17	0.28	0.65	0.10	0.94	0.04
**Icam1**	0.53	0.16	0.12	0.32	0.56	0.19	0.40	-0.44
**Nfkb1**	0.20	0.25	0.07	0.27	0.57	-0.14	0.88	-0.04
**Nfkb2**	0.12	1.03	0.06	0.65	0.36	1.16	0.53	-0.60

Figure S3

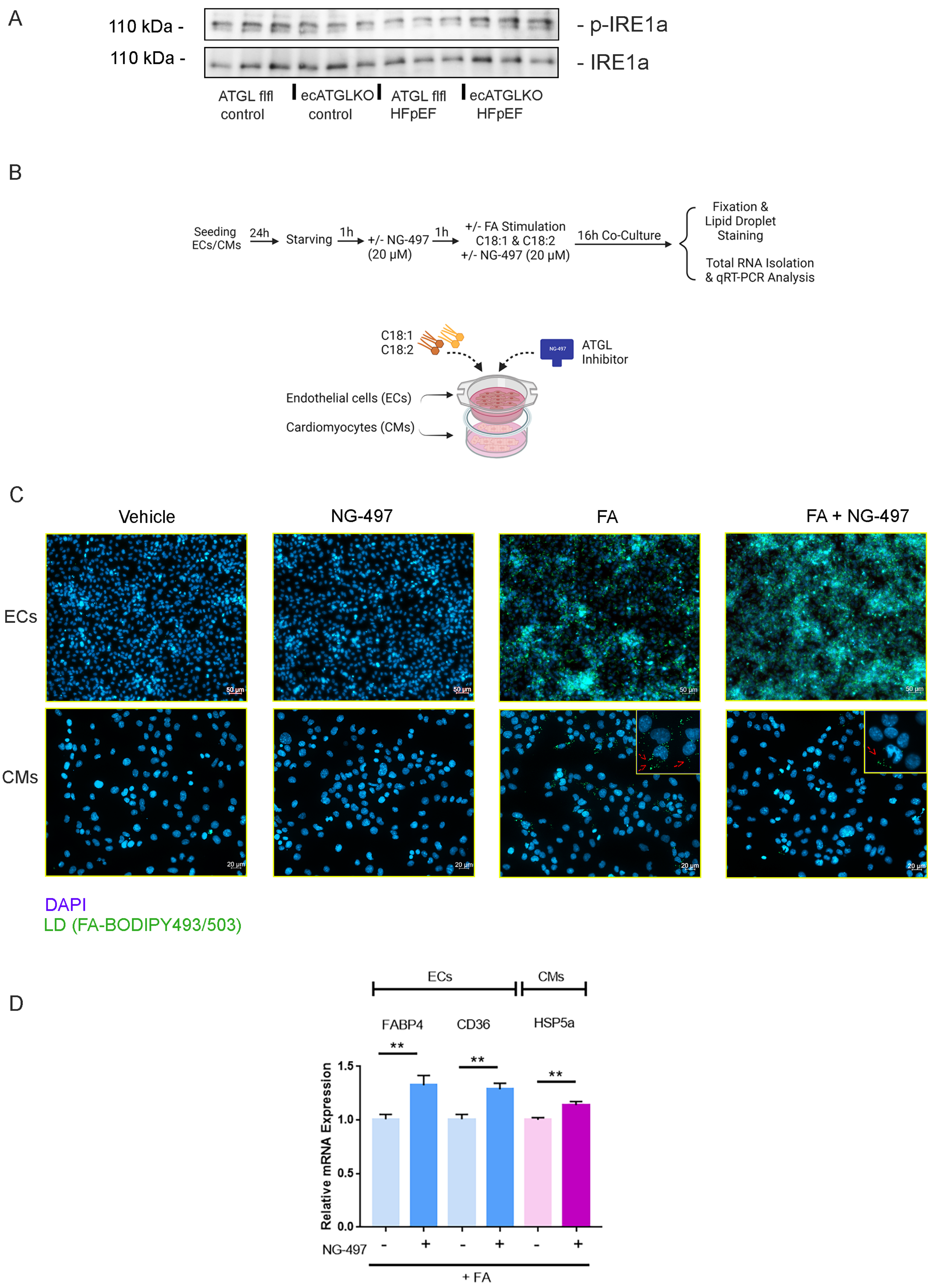
A
110 kDa -
110 kDa -
- p-IRE1a
- IRE1a
ATGL flfl control
ecATGLKO control
ATGL flfl HFpEF
ecATGLKO HFpEF
B
Seeding ECs/CMs
24h
Starving
1h
+/- NG-497 (20 µM)
1h
+/- FA Stimulation C18:1 & C18:2 +/- NG-497 (20 µM)
16h Co-Culture
Fixation & Lipid Droplet Staining
Total RNA Isolation & qRT-PCR Analysis
C18:1
C18:2
ATGL Inhibitor
Endothelial cells (ECs)
Cardiomyocytes (CMs)
C
Vehicle
NG-497
FA
FA + NG-497
ECs
CMs
50 µm
20 µm
DAPI
LD (FA-BODIPY493/503)
D
ECs
CMs
FABP4
CD36
HSP5a
**
**
**
Relative mRNA Expression
1.5
1.0
0.5
0.0
NG-497
-
+
-
+
-
+
+ FA

Figure S4
